# Probing Acidic and Defective Sites in Sulfated UiO-66 and ZrO_2_ via Adsorptive FTIR Spectroscopy

**DOI:** 10.3390/nano15110779

**Published:** 2025-05-22

**Authors:** Vera V. Butova, Olga A. Burachevskaia, Nikola L. Drenchev, Andrei A. Tereshchenko, Konstantin I. Hadjiivanov

**Affiliations:** 1 Institute of General and Inorganic Chemistry, Bulgarian Academy of Sciences, 1113 Sofia, Bulgaria; ndrenchev@svr.igic.bas.bg; 2 Academy of Biology and Biotechnology, Southern Federal University, 344090 Rostov-on-Don, Russia; 3 The Smart Materials Research Institute, Southern Federal University, 344090 Rostov-on-Don, Russia; oburachevskaya@sfedu.ru (O.A.B.); antereshenko@sfedu.ru (A.A.T.)

**Keywords:** MOF, functionalization, sulfation, UiO-66-SO_4_, ZrO_2_-SO_4_, S-ZrO_2_, benzoic acid, carbon monoxide, CD_3_CN, infrared spectroscopy

## Abstract

Sulfation is a common strategy to enhance the acidity and modify the adsorption properties of metal–organic frameworks (MOFs), yet its impact on the coordination and accessibility of active sites remains unclear. In this study, we investigate two structurally related systems—sulfated UiO-66 (UiO-66-SO_4_) and sulfated tetragonal zirconia (S-ZrO_2_)—by FTIR spectroscopy with probe molecules. Isotope exchange experiments on S-ZrO_2_ reveal that dehydration above 250 °C induces tridentate SO_4_ coordination, while hydration leads to a reversible transition to a bidentate coordination mode. In UiO-66-SO_4_, sulfates are coordinated in a bidentate fashion to Zr_6_O_6_ clusters, significantly affecting the accessibility of Zr sites in defective pores. This coordination prevents CO adsorption but allows acetonitrile adsorption even after room temperature activation. Unlike S-ZrO_2_, due to its lower thermal stability, UiO-66-SO_4_ cannot be evacuated at high temperatures and dehydration at 250 °C does not induce tridentate coordination. The presence of H-bonded hydroxyls in UiO-66-SO_4_ after activation at 250 °C supports this coordination model, indicating the formation of OH-coordinated Zr sites that are inaccessible to CO but interact with stronger bases like acetonitrile. Overall, this study provides new insights into the coordination chemistry of sulfated UiO-66 and highlights that sulfation can tune acidity and adsorption in MOFs for potential catalytic and adsorption applications.

## 1. Introduction

Zirconia (ZrO_2_) is extensively used in catalysis due to its acid–base properties and high thermal stability [1,2]. It serves as an active component in various catalytic processes, including hydrocarbon isomerization, alkylation, esterification, and oxidative dehydrogenation [3,4,5,6]. Moreover, zirconia is commonly employed as a support in heterogeneous catalysis, for example, in hydrodesulfurization catalysts, where it enhances the dispersion and stability of active metal species [7,8]. It is also incorporated into alumina-based catalysts in fluid catalytic cracking to improve thermal stability and acidity, enhancing overall catalytic performance [9,10]. At atmospheric pressure, ZrO_2_ exists in three different crystalline phases: monoclinic, tetragonal, and cubic. While the monoclinic phase is thermodynamically stable at room temperature, the tetragonal modification can be stabilized through doping or by reducing the particle size to the nanoscale [11,12,13]. The tetragonal phase, in particular, exhibits superior catalytic properties due to its higher surface energy, increased oxygen mobility, and enhanced acid strength. To further improve the catalytic activity of zirconia-based materials, surface modification strategies have been widely explored. One such approach involves the introduction of sulfate groups (SO_4_^2−^), which significantly enhance the surface acidity [14]. Sulfated zirconia (S-ZrO_2_) has been extensively studied and applied as a catalyst in various acid-catalyzed reactions, including the isomerization of alkanes, esterification, and biodiesel production [15,16].

UiO-66, a metal-organic framework (MOF) composed of Zr_6_O_4_(OH)_4_ clusters connected by terephthalate linkers, exhibits a structural similarity to tetragonal zirconia [17,18]. The ideal UiO-66 framework is highly stable due to the strong covalent bonding of each Zr_6_O_4_(OH)_4_ cluster with 12 organic linkers, resulting in a low concentration of accessible active sites. Consequently, pristine UiO-66 typically lacks significant catalytic activity. However, structural defects can be introduced during synthesis or post-synthetic treatment when some linkers are removed, leading to the formation of coordinatively unsaturated Zr sites within defective pores [19,20,21]. These Zr centers can act as Lewis acid sites and influence the material’s catalytic behavior.

In our previous work, we studied the accessibility of zirconium sites in defective UiO-66 and found that using benzoic acid as a modulator during synthesis promoted defect formation [20]. However, the resulting Zr sites were blocked by strongly bound benzoate anions, making them inaccessible. This material, referred to as UiO-66-BA, served as a reference for defective UiO-66 with blocked Zr sites. To make the Zr centers accessible, we applied a post-synthetic acid treatment that removed the benzoate anions. The resulting material, denoted as UiO-66-FA, retained the defective pore structure but had open Zr sites, making it suitable for further catalytic applications and post-synthetic modifications. In the present study, we use this approach to functionalize UiO-66 with sulfate groups and investigate their coordination and impact on active site accessibility.

Several studies have reported the incorporation of sulfate groups into zirconium-based MOFs, including UiO-66 (see examples in Table S1) [22,23,24,25,26,27,28,29]. For example, UiO-66 functionalized with sulfate groups demonstrated superior performance in gas-phase isobutene dimerization [22]. Similarly, the introduction of sulfate groups into MOF-808 resulted in the formation of a superacid due to the presence of Zr-bound sulfate species [23]. Treating UiO-66 with sulfuric acid led to simultaneous etching and the incorporation of SO_4_ groups, yielding materials that exhibited five times higher catalytic activity in the acetalization of benzaldehyde with methanol compared to untreated UiO-66 [30]. Despite the advantages that sulfation brings to UiO-66, the exact structure of sulfated materials remains unknown. The complexity of MOFs, composed of inorganic clusters and organic linkers, is further influenced by defects and guest molecules. For example, the IR spectrum of UiO-66 is dominated by linker bands, which obscure the identification of sulfate groups. Additionally, adsorbed water and residual solvents further complicate spectral analysis. Moreover, UiO-66 has lower thermal stability compared to zirconia, making some experimental approaches used for ZrO_2_ inapplicable for UiO-66.

Advanced characterization techniques such as X-ray photoelectron spectroscopy (XPS) and X-ray absorption near-edge structure (XANES) have been employed to study the structure and properties of S-ZrO_2_ [31,32,33]. In particular, infrared (IR) spectroscopy is a powerful tool for investigating the structural and chemical properties of solid surfaces. It enables the identification of surface functional groups, coordination environments, and adsorbed species, making it essential for understanding the behavior of catalysts and other functional materials [34,35]. However, the technique has limitations, particularly when it comes to detecting minor surface species or resolving overlapping vibrational bands. These challenges are especially pronounced when surface features are disordered or present at low concentrations.

An important example of surface modification where such complexities arise is sulfation, a widely used strategy to enhance the acidity and catalytic performance of materials. Even at low concentrations, sulfate groups can significantly influence the nature and accessibility of active sites, yet their detection and characterization can be difficult due to their weak or overlapping IR signals. To overcome these limitations, FTIR analysis can be complemented by advanced techniques such as the use of probe molecules, which interact selectively with specific surface sites, or isotopic substitution, which shifts vibrational bands and helps to distinguish between overlapping features. These approaches greatly improve the sensitivity and specificity of FTIR measurements, enabling a deeper understanding of how surface modifications influence material performance. Particularly, FTIR spectroscopy with probe molecules provided valuable insights into the nature of active sites and the interaction of sulfate groups with the zirconia surface [14,35,36]. Given the structural similarity between tetragonal zirconia and the inorganic clusters in UiO-66, findings from S-ZrO_2_ studies can offer useful insights into understanding the structure of sulfated UiO-66.

In this study, we apply IR spectroscopy to analyze the structure of S-ZrO_2_ and compare the obtained data with UiO-66-SO_4_. By probing the nature and accessibility of the active sites in these materials, we provide insights into their structural and catalytic properties.

## 2. Materials and Methods

### 2.1. Synthesis

The starting materials, zirconium tetrachloride (ZrCl_4_), zirconyl chloride (ZrOCl_2_), 1,4-benzene dicarboxylic acid (H_2_BDC), N, N-dimethylformamide (DMF), benzoic acid (BA), hydrochloric acid (37%), ammonium sulfate, ammonium hydroxide, acetone, and methanol, were purchased from Alfa Aesar (Ward Hill, MA, USA) and used without additional purification. Distilled water was purified via a Simplicity UV ultrapure water system (Millipore SAS, Molsheim, France).

Sulfated zirconia. Zirconia was prepared by adding 12% NH_3_ solution to 0.07 M ZrOCl_2_ solution until the pH reached 7. The resulting precipitate was filtered, thoroughly washed with water, dried, and calcined at 550 °C for 1 h. The obtained material was then suspended in 0.1 M H_2_SO_4_ for 20 h, washed with 0.01 M H_2_SO_4_, and finally calcined at 500 °C.

UiO-66-BA. We used the original procedure to obtain the UiO-66-BA sample [37]. Briefly, ZrCl_4_ was dissolved in DMF, and deionized water was added to the solution. BA was mixed in and stirred at room temperature until a clear solution was achieved. H_2_BDC was added and dissolved. The molar ratio of ZrCl_4_:H_2_BDC:H_2_O:DMF:BA was 1:1:3:300:10. The reaction mixture was then placed in an oven and heated at 120 °C for 24 h. After synthesis, a white precipitate formed and was collected by centrifugation. Then, it was washed twice with pure DMF and methanol. Finally, the washed precipitate was dried at 60 °C overnight to obtain the UiO-66-BA sample.

UiO-66-FA. We employed a modified procedure based on published data [20,38] for the post-synthetic treatment of the UiO-66-BA sample. In this process, 50 mg of UiO-66-BA sample was combined with 15 mL of DMF and 0.625 mL of 8M HCl. The mixture was stirred thoroughly and loosely covered with a watch glass. Subsequently, the flask and its contents were heated at 100 °C for 24 h in a preheated oven. After the treatment, a microcrystalline powder formed, which was separated from the mixture via centrifugation. The powder was then washed three times with fresh DMF. Lastly, the precipitate was separated from the solvent by centrifugation and dried overnight in an oven set to 60 °C. As shown below, formate ions were generated due to DMF hydrolysis, modifying the UiO-66-BA sample. Therefore, this modified sample is referred to as UiO-66-FA in the text.

UiO-66-SO_4_. Briefly, 50 mg of UiO-66-FA was mixed with ammonium sulfate solution (33 mg of (NH_4_)_2_SO_4_ in 2.5 mL of H_2_O). The suspension was mixed at room temperature for 72 h. After this, the precipitate was collected via centrifugation and washed twice with acetone. Finally, the powder was dried at 60 °C for 24 h.

### 2.2. Characterization

X-ray powder diffraction (XRD) patterns were recorded using a Bruker D2 PHASER diffractometer (Bruker AXS Inc., Fitchburg, WI, USA) over a 2θ range of 5–90°, with a step size of 0.01°, employing CuKα radiation (λ = 1.5417 Å). The obtained diffraction profiles were analyzed using Jana 2006 software (version 30/11/2014) [39].

Attenuated total reflection infrared (ATR-IR) spectra were acquired *ex situ* using a Bruker Vertex 70 spectrometer (Bruker Optik GmbH, Ettlingen, Germany). The measurements covered a spectral range of 4000–500 cm^−1^ with a resolution of 1 cm^−1^. Data collection was performed over 64 scans using an MCT detector and a Bruker Platinum ATR attachment (Bruker Optik GmbH, Ettlingen, Germany).

Raman spectroscopy was performed using Renishaw inVia Raman microscope (Enhanced Spectrometry Inc., Meridian, MS, USA) with a 532 nm (green) diode laser as the excitation source. Spectra were recorded within the 100–1700 cm^−1^ range and analyzed using Origin 9 software. Additionally, micro X-ray fluorescence (XRF) analysis was conducted using a Bruker M4 TORNADO 2D Micro-XRF spectrometer (Bruker, Billerica, MA, USA).

### 2.3. In Situ FTIR Experiments

*In situ* Fourier-transform infrared (FTIR) spectroscopy of self-supporting pellets was carried out using a Nicolet 6700 FTIR spectrometer (Thermo Fisher Scientific, Waltham, MA, USA), where each spectrum was obtained by accumulating 64 scans at a resolution of 2 cm^−1^. The pellets were prepared from sample powders and examined within a custom-designed IR cell, which allowed measurements at ambient conditions as well as at low temperature (−173 °C). The IR cell was connected to a vacuum adsorption system, maintaining a residual pressure below 10^−3^ Pa.

Activation. The S-ZrO_2_ sample was initially calcined in air at 470 °C for 3 h to remove potential organic impurities and then cooled to room temperature in an open vessel. It was subsequently evacuated during gradual heating from room temperature to 470 °C. The UiO-66 samples underwent stepwise evacuation under a dynamic vacuum, with heating from 25 °C to 250 °C. At selected temperatures, each sample was heated under a dynamic vacuum, cooled to room temperature, and then analyzed. After activation, the samples were rehydrated by exposure to 5 mbar of water vapor for 15 min, followed by evacuation at room temperature under a residual pressure below 10^−3^ Pa for 15 min.

Adsorption experiments. Before the adsorption studies, the activated and rehydrated samples were evacuated at 25 °C and then at 250 °C (for the UiO-66 samples) or 450 °C (for the S-ZrO_2_ sample). Adsorption experiments were performed by introducing 5 mbar of either CO (Merck, Darmstadt, Germany, 99.5% purity) or CD_3_CN (Merck, Darmstadt, Germany, deuteration degree 99.96%) into the IR cell. After exposure, the adsorbed gases were gradually diluted, followed by controlled step-wise evacuation. CO adsorption experiments were carried out at liquid nitrogen temperature, while CD_3_CN was adsorbed at room temperature.

Isotopic substitution. To substitute ^16^O with ^18^O in the S-ZrO_2_ sample, the material was first evacuated at 470 °C for 3 h to ensure complete dehydration. It was then exposed to 20 mbar of H_2_^18^O vapor (isotopic purity of 97 atom% ^18^O) at 470 °C for 3 h, followed by evacuation at the same temperature for 2 h. The sample was subsequently cooled under a dynamic vacuum, and its IR spectrum was recorded. This procedure was repeated to achieve a stepwise exchange of oxygen isotopes.

## 3. Results

### 3.1. Basic Characterization

**Figure 1:**
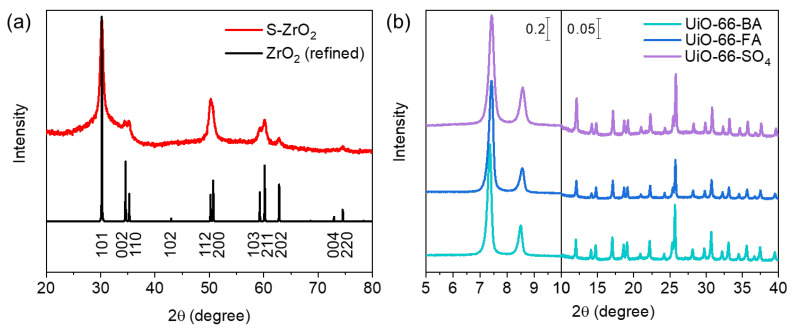
The experimental XRD pattern of the S-ZrO_2_ sample (**a**) and UiO-66 samples (**b**). The calculated powder pattern of ZrO_2_ with refined lattice constants is shown in black. Miller indices are provided below the calculated pattern for reference.

Powder XRD analysis revealed that the S-ZrO_2_ sample consisted of a pure tetragonal ZrO_2_ phase, with reflections fitting the P 42/nmc (137) space group (Figure 1a). The unit cell parameters were determined as follows: a = b = 3.5970(8) Å and c = 5.1841(15) Å (see Tables S2 and S3 for further details) [11,40,41,42]. Peak broadening analysis suggested that the sample consisted of nanoparticles with an average size of approximately 15 nm (Table S4).

The UiO-66-BA, UiO-66-FA, and UiO-66-SO_4_ samples exhibited a single-phase UiO-66 structure, as confirmed by XRD analysis (Figure 1b and Figure S1b, Tables S2 and S3) [43,44,45]. All reflections were indexed to a cubic symmetry with the space group Fm-3m. Despite undergoing post-synthetic treatments, the samples retained their structural integrity, showing no evidence of structural collapse or the formation of new phases.

**Figure 2:**
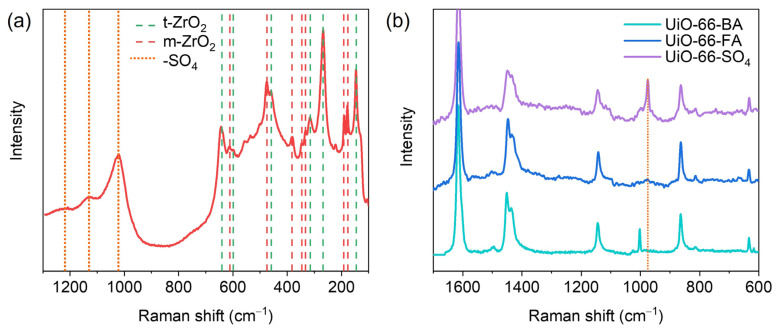
Raman spectra of S-ZrO_2_ (**a**) and UiO-66 samples (**b**). Dashed lines show bands assigned to ZrO_2_ phases (red for monoclinic and green for tetragonal), while orange dotted lines highlight bands assigned to SO_4_ groups.

The Raman spectrum of the S-ZrO_2_ sample is shown in Figure 2a. The most intense bands at 267, 315, 457, 600, and 644 cm^−1^ corresponded to the tetragonal ZrO_2_ phase. Additionally, the spectrum featured bands at 192, 335, 346, 382, 475, 612, and 636 (shoulder) cm^−1^, which were characteristic of the monoclinic ZrO_2_ phase [46]. While the presence of a minor monoclinic phase cannot be ruled out—possibly in an amorphous form or in an amount too small for XRD detection—the surface modification of zirconia with sulfate groups may also have contributed to these spectral features. Sulfation introduces structural distortions and surface species that can alter zirconia’s vibrational modes. The tetragonal phase has higher symmetry than the monoclinic phase, but sulfate coordination can lower this symmetry, producing vibrational characteristics similar to those of monoclinic ZrO_2_. Since these effects are surface-localized, they are detectable by Raman spectroscopy, a surface-sensitive technique, whereas XRD primarily reflects the bulk structure. Therefore, we concluded that the dominant phase in the S-ZrO_2_ sample was tetragonal, with surface-localized features of reduced symmetry likely arising from sulfation.

The Raman spectra of the UiO-66 samples aligned well with data reported in the literature [19,47]. In the case of the UiO-66-BA sample, an additional band at 1000 cm^−1^ was observed, which was attributed to benzoate ions. After treatment with HCl, these benzoate residues were removed, as evidenced by the absence of this band in the spectrum of the UiO-66-FA sample (Figure 2b).

Sulfate groups exhibit characteristic bands in Raman spectra, reflecting their vibrational modes: the S-ZrO_2_ sample showed three broad bands at 1020, 1135, and 1240 cm^−1^, and the UiO-66-SO_4_ sample displayed a sharp band at 975 cm^−1^ [48]. XRF analysis of the S-ZrO_2_ sample, conducted without any pre-treatment such as heating or evacuation, revealed an S:Zr ratio of approximately 0.08:1, confirming the presence of sulfate species. Similarly, the UiO-66-SO_4_ samples, also analyzed without pre-treatment, exhibited an S:Zr ratio of 0.4:1.

**Figure 3:**
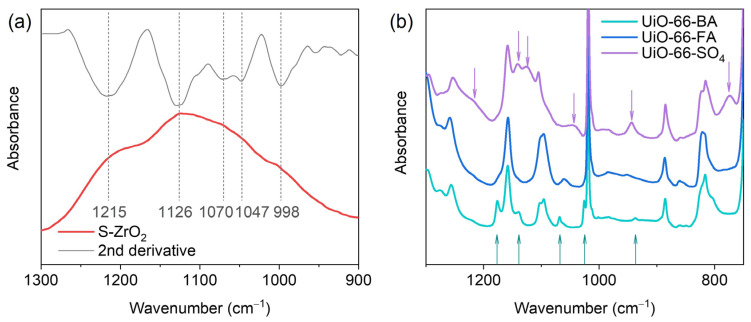
*Ex situ* IR spectra of the S-ZrO_2_ sample (**a**) and UiO-66 samples (**b**) recorded in ATR geometry. In (**a**), the gray curve represents the second derivative of the band in the S-ZrO_2_ spectrum attributed to SO_4_ groups. Its intensity was scaled by a factor of 15,000 for better visualization. In (**b**), blue arrows indicate bands associated with benzoate ions, while violet arrows mark bands corresponding to SO_4_ vibrations.

The IR spectrum of the as-prepared S-ZrO_2_ sample, recorded in ATR geometry, contained bands attributed to Zr–O bonds in ZrO_2_, as well as a band at 1620 cm^−1^ corresponding to water molecules [49,50] (Figure S2a). Figure 3a highlights the spectral region of S-ZrO_2_ associated with SO_4_ groups. A complex feature appeared in the 1300–900 cm^−1^ range, consisting of at least five bands. These bands were characteristic of surface-bonded SO_4_^2−^ ions, with a symmetric S–O stretching band at 998 cm^−1^ and asymmetric S–O stretching bands at 1047, 1070, 1126, and 1215 cm^−1^ [48,50].

The positions of the bands attributed to SO_4_ groups in the UiO-66-SO_4_ and S-ZrO_2_ spectra were similar, with only small shifts likely caused by adsorbed water molecules or linker effects (Figure S2b). Therefore, we propose that insights gained from studying sulfated zirconia can be applied to UiO-66-SO_4_. This approach aids in identifying key spectral features, despite strong linker bands overlapping with the fingerprint region in the IR spectrum.

In summary, the S-ZrO_2_ sample primarily consisted of tetragonal ZrO_2_ nanoparticles, with their surface decorated by SO_4_ groups, as confirmed by Raman, IR, and XRF analyses. XRD analysis verified that the UiO-66-BA, UiO-66-FA, and UiO-66-SO_4_ samples retained their single-phase UiO-66 structure despite undergoing post-synthetic treatments. The Raman and IR spectra aligned with the literature data, showing that UiO-66-BA exhibited a characteristic band from benzoate ions, which disappeared after HCl treatment in UiO-66-FA. Based on the analytical results and our recent findings, UiO-66-BA contained defective pores where zirconium ions were coordinated with benzoate residues [20]. Treatment with HCl in DMF led to the replacement of benzoate residues with formate ions. The hydrolysis of DMF in the presence of HCl resulted in formic acid formation, which contributed to the incorporation of formate species. Since formate ions are smaller and bind more weakly to Zr ions, thermal activation at 250 °C partially removed them, creating accessible Zr sites in defect pores within the UiO-66-FA sample. The introduction of sulfate groups into the UiO-66-FA sample, which contained accessible Zr ions in defect pores, led to the formation of UiO-66-SO_4_. The successful functionalization of UiO-66-FA with sulfate groups was confirmed by XRF, Raman, and IR spectroscopy analyses of the UiO-66-SO_4_ sample.

### 3.2. In Situ FTIR Background

**Figure 4:**
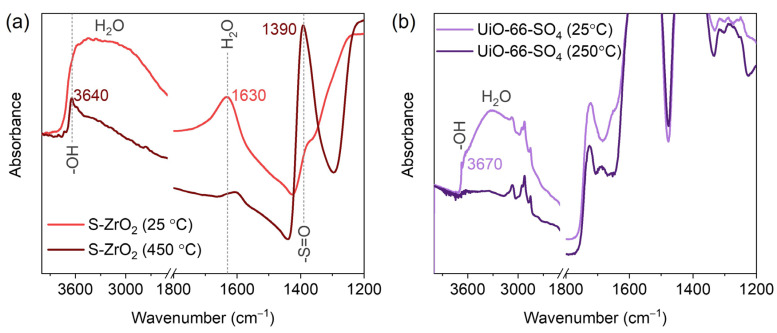
*In situ* FTIR spectra of the S-ZrO_2_ samples (**a**) and the UiO-66-SO_4_ samples (**b**). The spectra were recorded for samples that were activated, rehydrated, and subsequently evacuated at 25 °C (red spectrum in (**a**) and violet spectrum in (**b**)), as well as after additional evacuation at 250 °C (brown spectrum in (**a**) and dark violet spectrum in (**b**)).

Evacuation at different temperatures can influence surface properties by removing guest molecules. To study this effect, we conducted a stepwise evacuation of both S-ZrO_2_ and UiO-66-SO_4_ samples, tailoring the procedure to account for their differing thermal stabilities. For the *in situ* FTIR experiments, the S-ZrO_2_ sample was evacuated at the desired temperature up to 470 °C (Figure 4a and Figure S3 in SI). By contrast, UiO-66-SO_4_, which had lower thermal stability, was subjected to evacuation during gradual heating from 25 °C to 250 °C. This process effectively removed guest molecules from the MOF’s pores (see Figures S4 and S5 in SI). Following this, the sample was rehydrated in water vapor for 15 min at room temperature and then evacuated at the desired temperature for analysis.

After room temperature outgassing, both samples exhibited a broad band around 3345 cm^−1^ in their FTIR spectra due to ν(OH) modes of H-bonded hydroxyls (Figure 4a,b). At least part of this band was associated with adsorbed water molecules, as indicated by the appearance of δ(H_2_O) deformation modes at 1630 cm^−1^ in the spectra of the S-ZrO_2_ sample. Upon evacuation at higher temperatures, the intensity of these bands decreased significantly, indicating dehydration of the samples. Notably, the UiO-66-SO_4_ sample adsorbed more water than pristine UiO-66 and retained a significant amount of H_2_O even at 250 °C. Moreover, broad bands around 3300–3400 cm^−1^ in the spectrum of UiO-66-SO_4_ evacuated at 250 °C indicated the presence of H-bonded hydroxyl groups (Figure 4b and Figure S5). These bands were observed only in the spectrum of sulfated UiO-66, while the spectra of UiO-66-BA and UiO-66-FA did not exhibit such features. Thus, the presence of H-bonded hydroxyl groups in UiO-66-SO_4_ was the result of the sulfation process (see the Section 4 for more details).

As dehydration progressed, isolated OH groups became more pronounced. In the S-ZrO_2_ sample, this was indicated by the emergence of a band at 3640 cm^−1^, corresponding to OH groups [51,52]. The very broad band observed in the 2300–3300 cm^−1^ region of the S-ZrO_2_ spectrum was attributed to acidic protons formed due to the substitution of terminal OH groups with HSO_4_ anions [52]. For the UiO-66-SO_4_ sample, characteristic μ_3_-OH bands were observed at 3670 cm^−1^ after evacuation at room temperature but disappeared upon heating to 250 °C. Notably, this process was reversible, as rehydration with water vapor restored the intensity of the OH band.

The bands associated with sulfate groups in UiO-66-SO_4_ overlapped with those of the linker, complicating their analysis. However, in the case of S-ZrO_2_, the effects of dehydration were clearly observable. Specifically, the sulfate band at around 1200 cm^−1^ shifted to 1390 cm^−1^ after dehydration [50].

### 3.3. Oxygen Isotopic Exchange

Sulfate groups contain four oxygen atoms and can bond to the zirconia surface through one, two, or three of them [46,49,52,53]. Consequently, depending on the coordination mode, sulfate groups on the surface of the S-ZrO_2_ sample may have had one, two, or three unbonded oxygen atoms. To investigate the bonding configuration of the SO_4_ groups, we applied ^18^O → ^16^O isotopic substitution.

**Figure 5:**
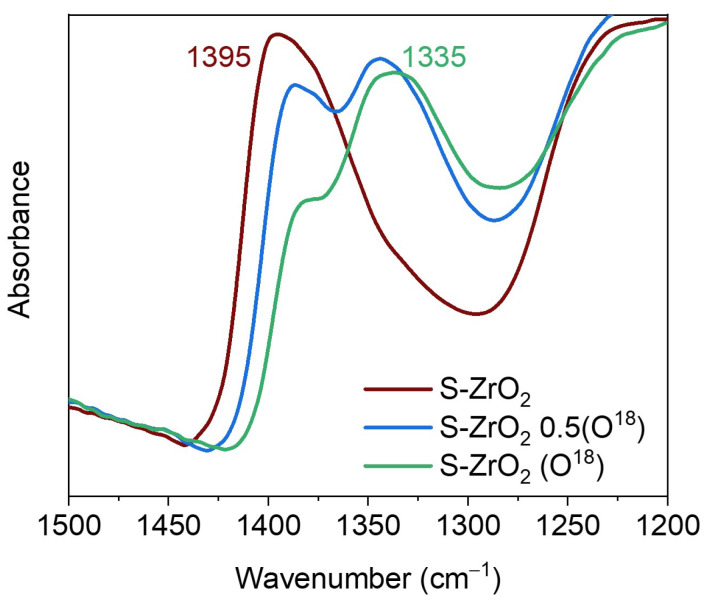
*In situ* FTIR spectra of the S-ZrO_2_ sample after evacuation at 470 °C (brown), after interaction with H_2_^18^O (20 mbar) at 470 °C for 3 h followed by evacuation at the same temperature (blue), and after repeating the treatment with H_2_^18^O (green). Spectra are provided in the 1500–1200 cm^−1^ region.

Figure 5 shows the spectra of activated S-ZrO_2_ before and after the isotopic exchange. After the first ^18^O → ^16^O substitution procedure, the band at 1395 cm^−1^ declined and partly shifted to 1385 cm^−1^. At the same time, a new band emerged at 1345 cm^−1^. The shift coincided well with the theoretical expectations for an S–O bond. The second isotopic exchange resulted in additional erosion and a red shift of the band at 1395 cm^−1^, as well as the further development of the lower-frequency band. Noticeably, no intermediate band was observed. Therefore, our results unambiguously proved that the 1395 cm^−1^ band corresponded to isolated S=O moieties, which was consistent with a tridentate coordination of the sulfate ion in the S-ZrO_2_ sample (see more details in the Section 4).

### 3.4. CO Adsorption

Carbon monoxide (CO) is a widely used probe molecule in IR experiments because of its weak basic properties, which make it effective for identifying acidic sites on solid surfaces [36,54]. In this study, we employed CO as a probe to investigate the S-ZrO_2_ and UiO-66 samples.

**Figure 6:**
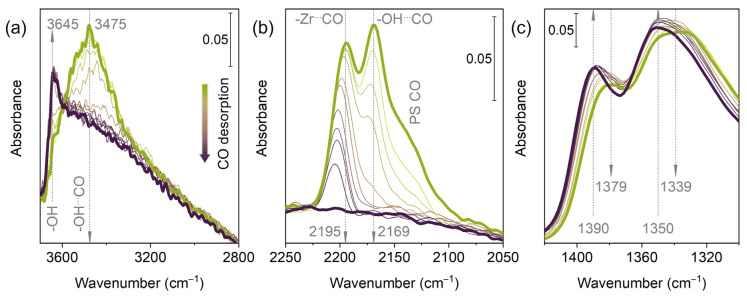
*In situ* FTIR spectra of the S-ZrO_2_–^18^O sample after the introduction of CO (5 mbar), followed by stepwise evacuation. Before the experiment, the sample was evacuated at 450 °C. Spectra are provided in the 3650–2800 cm^−1^ (**a**), 2250–2050 cm^−1^ (**b**), and 1420–1300 cm^−1^ (**c**) regions. PS stands for physisorbed.

Figure 6 represents the IR spectrum of the S-ZrO_2_-^18^O sample obtained during CO desorption. Two main bands at 2195 and 2169 cm^−1^ appeared together with an ill-defined shoulder around 2138 cm^−1^. In the OH region, the band at 3645 cm^−1^ almost disappeared, and a new, broader, and more intense band at 3475 cm^−1^ emerged, evidencing OH–CO interaction. The shoulder at 2138 cm^−1^ was very sensitive to the equilibrium pressure and quickly disappeared at low pressures. Consequently, it was attributed to physically adsorbed CO. The next band to disappear upon evacuation was that at 2169 cm^−1^. It changed in concert with the OH band at 3475 cm^−1^, which allowed it to be attributed to OH⋯CO adducts. The CO-induced shift of the OH modes was 170 cm^−1^. By comparison, non-sulfated ZrO_2_ showed a smaller OH band shift of about 90 cm^−1^ upon CO adsorption [55]. The larger shift observed in the sulfated samples indicated that sulfation enhanced the acidity of the OH groups, resulting in stronger interactions with CO. The band at 2195 cm^−1^ was attributed to CO adsorption on Zr^4+^ sites. Notably, this band was observed only after the S-ZrO_2_ sample was activated at 450 °C, while no such band appeared for the sample activated at room temperature (SI Figure S6). This difference was due to the presence of adsorbed water molecules on the sample evacuated at room temperature, which remained coordinated to Zr^4+^ sites and blocked CO adsorption. High-temperature activation led to dehydration of the surface, exposing previously inaccessible Zr^4+^ sites and enabling CO adsorption.

In the IR spectrum of the S-ZrO_2_–^18^O sample, bands at 1390 and 1350 cm^−1^ were attributed to sulfate groups. Upon exposure to CO, these bands gradually shifted to 1379 and 1339 cm^−1^. A similar effect has been reported for sulfated titania [56]. This shift of the ν(S–O) modes upon CO adsorption was likely due to electronic inductive effects.

**Figure 7:**
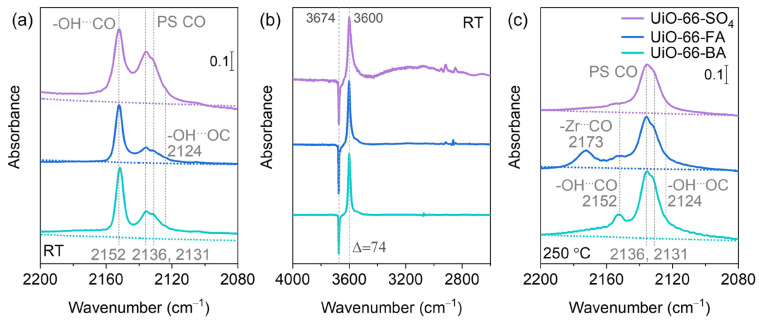
*In situ* FTIR spectra recorded after low-temperature CO adsorption (3 mbar equilibrium pressure) on UiO-66-BA, UiO-66-FA, and UiO-66-SO_4_ samples evacuated at 25 °C (**a**,**b**) and at 250 °C (**c**). Part (**b**) shows the difference spectrum, highlighting changes before and after CO exposure (1 mbar) at liquid nitrogen temperature for samples evacuated at room temperature. The intensities were scaled by factors of 0.5 for UiO-66-FA and 0.9 for UiO-66-BA to improve comparability, as tablet thickness was not consistent across the experiments. PS stands for physically adsorbed.

After room temperature activation, all three UiO-66 samples—UiO-66-BA, UiO-66-FA, and UiO-66-SO_4_—contained μ_3_-OH groups with Brønsted acidic properties. Adsorption of CO onto the μ_3_-OH groups of Zr_6_O_4_(OH)_4_ clusters in all UiO-66 samples resulted in the appearance of a C–O stretching band at 2152 cm^−1^ (Figure 7a). This band corresponded to the stretching vibration of CO molecules hydrogen-bonded to the μ_3_-OH group via the carbon atom (μ_3_-OH⋯C≡O). Additionally, bands at 2131 and 2136 cm^−1^ were attributed to physically adsorbed CO [21]. A weak band at 2124 cm^−1^, assigned to CO bonded to the μ_3_-OH group via its oxygen atom (μ_3_-OH⋯O≡C), was also detected [21,57]. The much lower intensity of this band compared to the μ_3_-OH⋯C≡O adducts showed that CO preferentially formed hydrogen bonds through its carbon atom. CO adsorption also caused a shift of the μ_3_-O–H stretching band (Figure 7b), confirming the formation of μ_3_-OH⋯C≡O adducts. All three UiO-66 samples exhibited a similar shift of approximately 74 cm^−1^ upon CO adsorption.

A careful inspection of the spectra revealed a low-intensity, broad band extending from 3400 to 2900 cm^−1^, which appeared only with the UiO-66-SO_4_ sample (Figure 7b). This band indicated the presence of H-bonded hydroxyls. The corresponding negative band was likely unidentifiable due to its low intensity and broad linewidth. In any case, this band suggested the existence of OH groups with enhanced acidity, which were likely associated with the presence of sulfate groups. However, the intensity of this band was not high, as most of the strongly acidic hydroxyls were already involved in H-bond formation before CO adsorption.

Evacuating the samples at 250 °C led to a significant reduction in the intensity of the μ_3_-OH bands around 3674 cm^−1^, suggesting that most of the Zr_6_O_4_(OH)_4_ clusters had been converted to Zr_6_O_6_ (Figure S7 in SI). Figure 7c presents the spectra of CO adsorbed on UiO-66-BA, UiO-66-FA, and UiO-66-SO_4_ after evacuation at 250 °C. At this stage, the most intense bands corresponded to physically adsorbed CO (2131 and 2136 cm^−1^) [21]. Low-intensity bands at 2152 cm^−1^ were still visible, indicating residual μ_3_-OH groups. No prominent peaks were detected in the 3500–3700 cm^−1^ region in the UiO-66-SO_4_ spectrum, likely due to the signal being at the noise level (Figure S7 in SI).

The spectrum of CO adsorbed on the UiO-66-FA sample revealed a new band at 2173 cm^−1^, which was absent in the spectra of the UiO-66-BA and UiO-66-SO_4_ samples. This band was attributed to CO interacting with coordinatively unsaturated Zr^4+^ sites in defect pores [21,57] (see Section 4).

### 3.5. CD_3_CN Adsorption

Compared to carbon monoxide, acetonitrile exhibits a stronger basic character, making it particularly suitable for probing acidic surface sites. In our experiments, we used deuterated acetonitrile (CD_3_CN) instead of regular acetonitrile (CH_3_CN) to avoid the splitting of C–N vibrational modes due to Fermi resonance [36].

**Figure 8:**
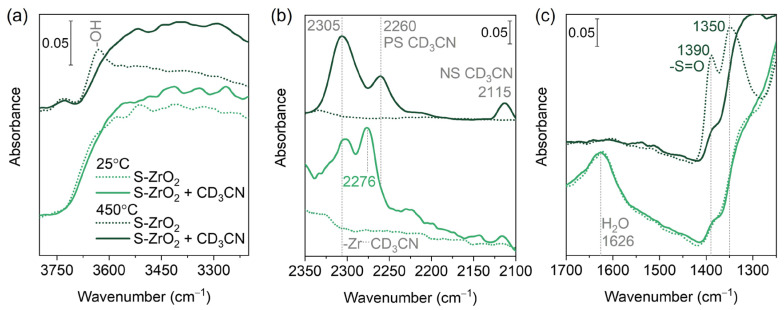
*In situ* IR spectra of the S-ZrO_2_–^18^O sample before (dotted) and after (solid) exposure to 1 mbar of CD_3_CN. Prior to recording the spectra, the S-ZrO_2_ sample was treated twice with H_2_^18^O and evacuated at 25 °C (light green) or 450 °C (dark green). Spectra are provided in the 3800–3200 cm^−1^ (**a**), 2350–2100 cm^−1^ (**b**), and 1700–1250 cm^−1^ (**c**) regions. PS stands for physisorbed; NS designates non-specific.

Figure 8a presents the OH region of S-ZrO_2_ samples after vacuum thermal activation (dotted lines) and after exposure to 1 mbar of CD_3_CN (solid lines). Additional spectra recorded during evacuation from 5 mbar are provided in the Supporting Information. After room temperature activation, the S-ZrO_2_ sample contained a significant amount of physisorbed water, as indicated by a broad band in the 3200–3600 cm^−1^ region and a band at 1626 cm^−1^ (see the light green lines in Figure 8). Evacuation at 450 °C removed most of the physisorbed water, revealing a band at 3630 cm^−1^, which corresponded to OH groups (see dark green lines in Figure 8). Upon CD_3_CN adsorption, the 3630 cm^−1^ band shifted, confirming the Brønsted acidity of the corresponding hydroxyls, as they formed hydrogen bonds with CD_3_CN.

CD_3_CN adsorption on the hydrated sample hardly affected the S–O bands (Figure 8c), suggesting that, in this case, the symmetry of the sulfate anions was practically preserved. By contrast, drastic changes were observed for the activated sample. In particular, the high-frequency S=O stretching band shifted downward by approximately 60 cm^−1^, indicating a change in symmetry. The spectra suggested that the state of the sulfates after CD_3_CN adsorption was similar to that of the hydrated sample.

In the νCN region, CD_3_CN adsorption on S-ZrO_2_ resulted in the appearance of several new bands (Figure 8b). The band at 2260 cm^−1^ corresponded to hydrogen-bonded and/or physisorbed CD_3_CN. The band at 2115 cm^−1^ was assigned to the ν_s_(CD_3_) vibration of all adsorbed acetonitrile species. Bands at 2300–2305 cm^−1^ indicated the coordination of CD_3_CN to Zr^4+^ Lewis acid sites, observed on both room temperature and 450 °C–activated S-ZrO_2_ samples. The band at 2275 cm^−1^ appeared only for hydrated S-ZrO_2_ samples and was associated with OH groups exhibiting medium-low Brønsted acidity [51]. These OH species were linked to hydrated sulfate groups, which contributed to the overall surface acidity.

**Figure 9:**
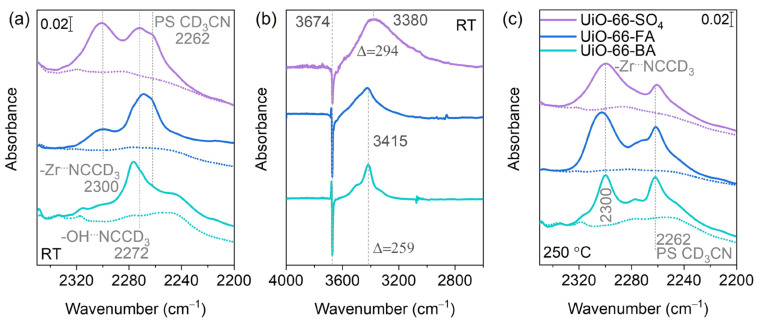
*In situ* FTIR spectra recorded after room temperature CD_3_CN adsorption (2 mbar equilibrium pressure) on UiO-66-BA, UiO-66-FA, and UiO-66-SO_4_ samples evacuated at 25 °C (**a**,**b**) and at 250 °C (**c**). Part (**b**) shows the difference spectrum, highlighting changes before and after CD_3_CN exposure (2 mbar) for samples evacuated at room temperature. PS stands for physisorbed.

Figure 9a presents the spectra of CD_3_CN adsorbed on UiO-66 samples activated at room temperature (see also Figure S8a). The spectra of all three UiO-66 samples exhibited a band at 2262 cm^−1^, which corresponded to the C≡N stretching mode of physically adsorbed acetonitrile. The adsorption of CD_3_CN on μ_3_-OH groups in Zr_6_O_4_(OH)_4_ clusters gave rise to a band at approximately 2272 cm^−1^ [20,21,54].

The interaction between μ_3_-OH groups and CD_3_CN was further evidenced by a red shift of the μ_3_-OH band upon CD_3_CN exposure (Figure 9b). This shift, which was greater than that observed for CO adsorption, highlighted the stronger basicity of acetonitrile. Moreover, the shift for the UiO-66-SO_4_ sample was larger, and the shifted band included components at lower frequencies, suggesting an additional fraction of more acidic hydroxyls. This was in general agreement with the results obtained with CO as a probe, indicating the existence of some OH groups on the sulfated sample that were more acidic than the μ_3_-OH groups.

Additionally, a band at 2303 cm^−1^ appeared in the spectra of the UiO-66-FA and UiO-66-SO_4_ samples (Figure 9a), signifying CD_3_CN interaction with Zr^4+^ ions. This band was nearly absent in the spectrum of UiO-66-BA, suggesting that these adsorption sites on the hydrated sample corresponded to bare Zr^4+^ ions located in defect pores, created by the removal of modulator anions or affected by sulfate species. Under normal conditions, such sites would be blocked by adsorbed water. However, due to its relatively high proton affinity (779.2 kJ mol^−1^) compared to H_2_O (691 kJ mol^−1^) [19,35], acetonitrile can effectively replace pre-adsorbed water. Notably, the spectrum of the UiO-66-SO_4_ sample displayed a more intense band at 2303 cm^−1^, suggesting a greater abundance of these exposed Zr^4+^ sites.

Following activation at 250 °C, dehydration and the loss of μ_3_-OH groups occurred, resulting in only weak peaks at 2272 cm^−1^ (Figure 9c and Figure S8b). Meanwhile, bands in the 2300–2305 cm^−1^ region became more prominent in all three samples. In our previous studies, we attributed these bands to CD_3_CN adsorption on bare Zr^4+^ sites within regular pores [20].

UiO-66-SO_4_ and S-ZrO_2_ exhibited notable similarities in their interaction with CD_3_CN. In their hydrated states, both materials adsorbed acetonitrile through interactions with surface OH groups and Zr sites. This behavior contrasted with CO adsorption, as the higher basicity of acetonitrile enabled it to displace adsorbed water molecules and access Zr sites. Upon dehydration, only residual OH groups remained, and both materials adsorbed CD_3_CN primarily through coordination to Zr^4+^ ions. Notably, the IR bands associated with Zr⋯CD_3_CN interactions appeared in the same spectral region (2300–2305 cm^−1^) for both UiO-66-SO_4_ and S-ZrO_2_, indicating similar acidity of the Zr^4+^ sites. Additionally, dehydrated S-ZrO_2_ showed clear spectral changes related to CD_3_CN interaction with surface sulfate groups. By contrast, no similar changes were noticed with the hydrated S-ZrO_2_ and UiO-66-SO_4_.

## 4. Discussion

**Figure 10:**
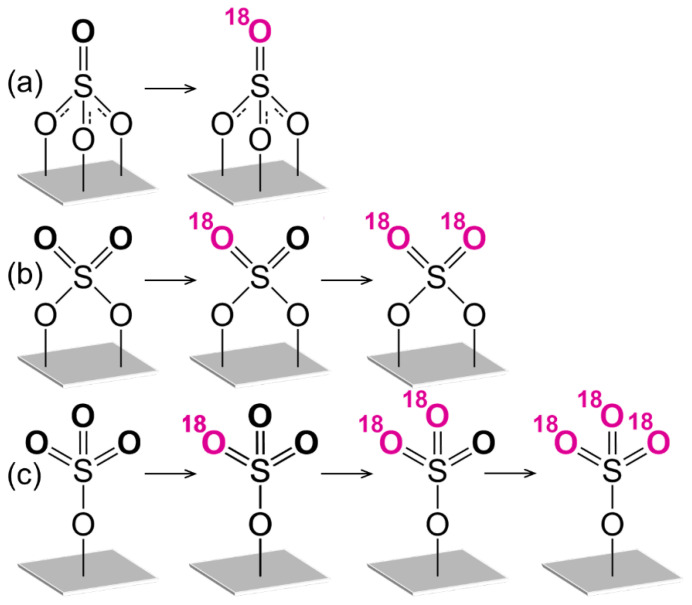
Stepwise exchange of ^16^O by ^18^O in different possible surface sulfates. Part (**a**) illustrates the tridentate coordination of SO_4_, part (**b**) depicts bidentate coordination, and part (**c**) demonstrates the monodentate binding variant of SO_4_. The zirconium oxide surface is schematically represented by gray rectangles.

In this study, we compared tetragonal sulfated zirconia (S-ZrO_2_) and sulfated UiO-66. While these materials belong to different classes, they share a common structural motif—SO_4_^2−^ connected to Zr-O clusters. Observing trends in ZrO_2_ can provide valuable insights into the behavior of UiO-66. This MOF is well known for its structural tolerance to defects, which significantly influences its properties. To explore these effects, we used three UiO-66 samples (Figure 10):

- UiO-66-BA—contains defects, but they are coordinated with benzoate ions, making the Zr sites inaccessible.
- UiO-66-FA—the benzoate ions have been removed, exposing Zr sites in defect pores.
- UiO-66-SO_4_—derived from UiO-66-FA by modifying it with sulfate (SO_4_) groups.

A central question in the study of sulfated zirconia concerns the coordination mode of SO_4_ groups on the zirconia surface. While some reports attribute the observed vibrational bands to bidentate bridging coordination, others suggest the presence of monodentate or tridentate configurations [46,49,52,53]. This discrepancy arises from the inherent complexity of solid surfaces modified with sulfate groups, where the ions may be unevenly distributed and exhibit multiple coordination modes simultaneously.

In its free state, the sulfate ion (SO_4_^2−^) exhibits T_d_ symmetry, with four equivalent S–O bonds arranged in a perfect tetrahedral geometry [58]. This high symmetry gives rise to four fundamental vibrational modes: two stretching modes—symmetric (ν_1_) and antisymmetric (ν_3_)—and two bending modes, also symmetric (ν_2_) and antisymmetric (ν_4_) [58,59].

The bending vibrations (ν_2_ and ν_4_) typically appear in the 680–450 cm^−1^ region. However, in vibrational studies involving solid surfaces, these low-frequency modes are often obscured by strong absorptions from the substrate itself and thus are generally not considered useful for surface vibrational spectroscopy.

The S–O stretching modes are of greater importance in such studies. In the free ion, selection rules dictate that the ν_1_ mode is only Raman-active, while the ν_3_ mode is also IR-active, appearing at 1104 cm^−1^. Upon coordination of sulfate ions to metal cations, molecular symmetry is reduced—commonly to C_3v_, C_2v_, or C_s_—relaxing the selection rules. As a result, the ν_1_ mode becomes IR-active, and the degenerate ν_3_ mode splits into two or more components, depending on the coordination [59].

Coordination also causes shifts in the vibrational frequencies. Monodentate coordination generally results in minor frequency shifts and limited band splitting. By contrast, bidentate coordination—either bridging or chelating—leads to more significant shifts and pronounced splitting, reflecting the stronger perturbation of the sulfate structure. While tridentate coordination of sulfate anions has not been reported in bulk crystalline compounds, if present, it would likely lead to a further increase in S–O stretching frequencies due to enhanced bonding asymmetry.

Organic sulfate esters, by contrast, exhibit distinct vibrational features arising from their characteristic O=S=O moiety. These species typically show symmetric and asymmetric S–O stretching bands in the 1450–1350 cm^−1^ and 1230–1150 cm^−1^ regions, respectively [60].

Representative examples of bulk sulfates and their vibrational signatures are summarized in Table 1.

The high-frequency band at 1390 cm^−1^ observed in the spectrum of dehydrated S-ZrO_2_ is close to that typically found in sulfate esters, which initially supported the hypothesis of bidentate coordination of the sulfate anion on the zirconia surface. However, this band may also correspond to isolated S=O stretching modes associated with tridentate sulfate species (see Figure 10). To determine the coordination mode in our S-ZrO_2_ sample, we performed ^16^O to ^18^O isotope exchange and monitored the changes using FTIR spectroscopy. If the band around 1390 cm^−1^ characterized an isolated S=O bond, it should be shifted directly by a factor of 0.98 (characteristic of the S–O bond). However, with a bidentate structure, a two-step shift should be observed with the appearance of an intermediate band corresponding to ^18^O-S-^16^O moieties (Figure 10b). Similar is the case with monodentate species, where, however, two intermediate bands should be observed. We observed a shift of the band at 1390 cm^−1^ without splitting, indicating the tridentate coordination of the SO_4_ groups.

**Scheme 1:**
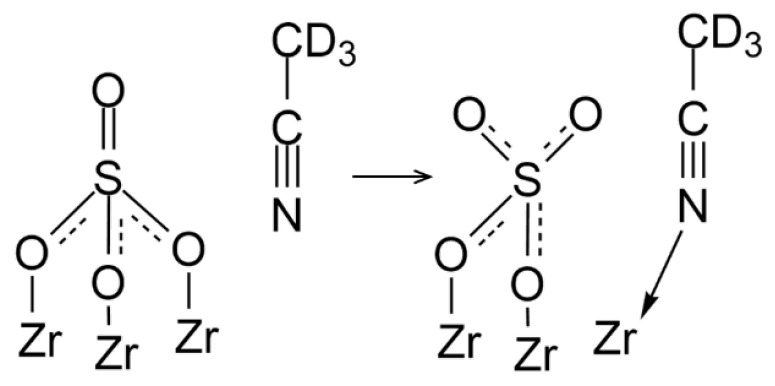
Effect of CD_3_CN adsorption on the surface of S-ZrO_2_.

After adsorption of CD_3_CN on Lewis acid sites of sulfated zirconia, the band at 1390 cm^−1^ shifted to lower frequencies. This shift occurred simultaneously with the formation of CD_3_CN-Zr^4+^ complexes, indicating the occupation of Lewis acid sites. These observations suggest that the coordination of acetonitrile to Zr^4+^ induces significant changes in the S=O bond. A similar effect was previously reported in the literature, where sulfate rearrangement was proposed as a possible explanation [56]. These findings can be interpreted based on the assumption that acetonitrile adsorption on the Zr^4+^ site triggers a structural rearrangement of the sulfate group, as illustrated in Scheme 1.

CO, being a weaker base, cannot induce significant structural changes in sulfate groups and only causes a slight shift of the S=O band. However, the presence of this effect suggests that at least one of the Zr^4+^ sites bound to the sulfate anion is coordinatively unsaturated and can function as a Lewis acid site. By contrast, acetonitrile, a much stronger base than CO, facilitates the breaking of one S–O–Zr bond.

**Scheme 2:**
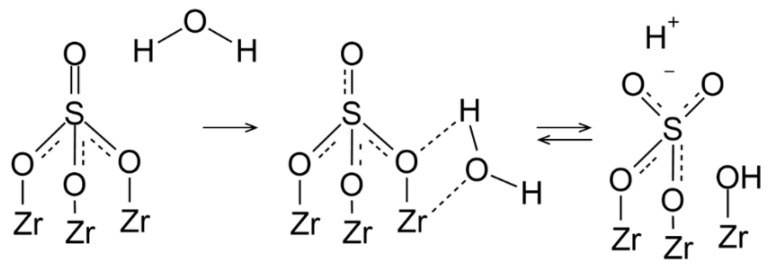
Proposed scheme illustrating the interaction of the S-ZrO_2_ surface with adsorbed water.

Additionally, we observed a red shift of the 1390 cm^−1^ band associated with S=O vibrations upon hydration. In the spectrum of the hydrated S-ZrO_2_ sample, multiple bands emerged in the 1000–1200 cm^−1^ region. This effect closely resembled that observed upon CD_3_CN adsorption, though the red shift appeared more pronounced. Based on this observation, we propose the following scheme for the process, where an OH group and a delocalized proton are formed (Scheme 2). Notably, the OH group is also involved in hydrogen bonding (not depicted in Scheme 2), as no band corresponding to isolated hydroxyls was observed in the IR spectra.

The proposed structure of sulfates on the hydrated S-ZrO_2_ is also supported by the IR and Raman spectra shown in Figure 2 and Figure 3. A comparison with the data presented in Table 1 shows that the spectra are consistent with bidentate coordination. The proposed scheme also aligns with the observation that CO does not adsorb on the hydrated sample. Moreover, bidentate coordination of sulfate to Zr clusters was observed by single-crystal XRD in MOF-808, which featured the same Zr node structure [23]. NU-1000 exhibited a dynamic sulfate binding motif that transitions from monodentate to bidentate and eventually to tridentate coordination, depending on the degree of hydration [28]. This behavior was confirmed by single-crystal XRD and supported by DFT calculations.

**Figure 11:**
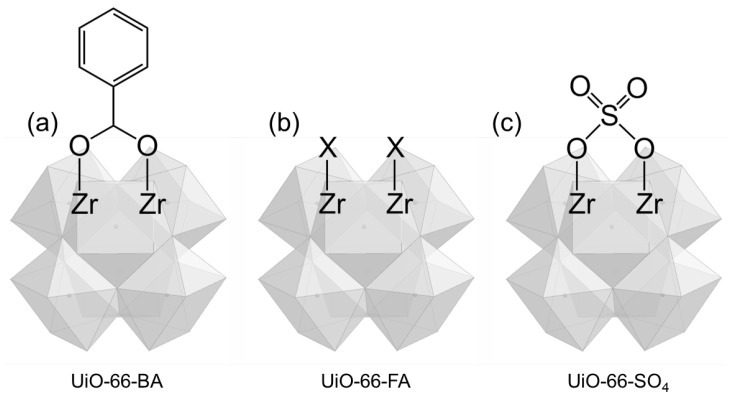
Schematic representation of UiO-66-BA (**a**), UiO-66-FA (**b**), and UiO-66-SO_4_ (**c**) samples. The figure provides a schematic view of three Zr ions within a defective pore. Light gray polyhedra illustrate the coordination of Zr with oxygen. In part (**c**), ion X represents a temporary ion compensating for charge after post-synthetic treatment and activation.

To better understand the structure of UiO-66-SO_4_, we begin by comparing it with its UiO-66-BA and UiO-66-FA counterparts (Figure 11). For our *in situ* studies, we combined two probe molecules—CO and CD_3_CN.

Carbon monoxide is a very weak base. When adsorbed at a low temperature, it probes the state of active sites without inducing structural rearrangements. CO adsorption revealed the absence of accessible Zr^4+^ sites on the pristine UiO-66-BA sample, even after evacuation at 250 °C (Table 2). This indicated that these sites were coordinatively saturated—bonded to terephthalate ligands in regular pores and to benzoate ions in defective pores (Figure 11a). By contrast, CO adsorption on the activated UiO-66-FA sample revealed the presence of Zr^4+^ Lewis acid sites associated with cations in defective pores, which become accessible after removal of a temporary ligand (Figure 11b). These sites were not detected in the unactivated sample because they were blocked by water molecules. Similarly, for UiO-66-SO_4_, no accessible Zr^4+^ sites were observed, indicating a situation similar to that of UiO-66-BA (Figure 11c). CD_3_CN is a stronger base than CO and may induce structural rearrangements to form stable complexes. It can also displace pre-adsorbed water. As previously reported [20,54], CD_3_CN detects Zr^4+^ sites in regular pores, but only after the μ_3_-OH groups are removed by sample dehydroxylation. Evidently, the same situation applied to the Zr^4+^ sites from the defective pores in the UiO-66-BA sample (Table 2).

Adsorption of CD_3_CN on the UiO-66-FA sample revealed the presence of Lewis acid sites even on the 25 °C evacuated sample. Since no such sites were observed with UiO-66-BA, we could confidently attribute them to Zr^4+^ cations located in defective pores. These sites were not detected by CO because they were blocked by H_2_O molecules. However, as previously noted, CD_3_CN is capable of displacing pre-adsorbed water. The number of Lewis acid sites increased following activation at 250 °C, as CD_3_CN then also interacted with Zr^4+^ cations in dehydroxylated Zr clusters within the regular pores.

With UiO-66-SO_4,_ we found Lewis acid sites even with the sample activated at 25 °C, which was an important difference compared to the UiO-66-BA sample. It was evident that the inorganic cluster in the sulfated sample was more prone to reconstruction, which was most likely due to the ionic nature of the Zr–sulfate bond.

To evaluate how SO_4_ groups coordinate within the UiO-66 framework, we compared these findings with the behavior of S-ZrO_2_. The experimental results revealed similarities between UiO-66-SO_4_ and hydrated S-ZrO_2_—both samples exhibited accessible Zr sites for acetonitrile but not for CO. Based on this, we propose that SO_4_ groups in UiO-66-SO_4_ are coordinated in a bidentate fashion to the Zr_6_O_4_(OH)_4_ clusters.

In the S-ZrO_2_ sample, dehydration led to tridentate coordination, while rehydration resulted in the breaking of one S–O–Zr bond, reverting to bidentate SO_4_ bonding. This process was reversible in S-ZrO_2_. However, the less stable UiO-66 framework did not allow for reproduction of this process, as activation at 450 °C (the temperature used for S-ZrO_2_) would lead to structural collapse. The presence of OH bands in the spectrum of UiO-66-SO_4_ after activation at 250 °C further supported this proposed coordination scheme (Figure S5).

**Scheme 3:**
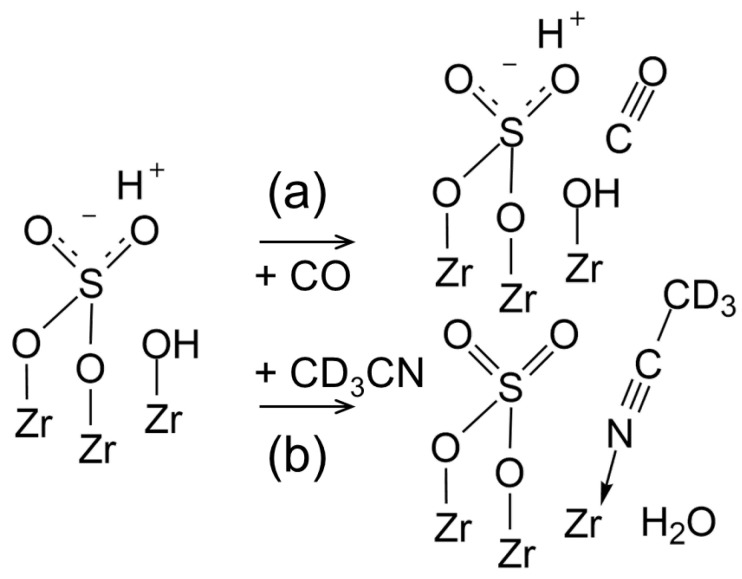
Proposed scheme illustrating the interaction of the UiO-66-SO_4_ structure with CO (**a**) and CD_3_CN (**b**). See the text for details.

Thus, UiO-66-SO_4_ contains Zr sites in defective pores, which are linked to OH groups formed due to bidentate SO_4_ coordination. These Zr sites are inaccessible to CO because CO, being a weak base, cannot displace OH groups (Scheme 3a). By contrast, acetonitrile, as a stronger base, is capable of replacing OH groups (forming H_2_O with H^+^) and binding to Zr in defective pores (Scheme 3b).

## 5. Conclusions

In this study, we investigated the effect of sulfation on the structural and adsorption properties of UiO-66 using FTIR spectroscopy with probe molecules. To better understand sulfate coordination, we used sulfated tetragonal zirconia as a model system, given its structural similarity to Zr_6_O_6_ clusters in UiO-66. However, even for sulfated zirconia, the precise coordination of sulfate groups remains an open question. Through isotope exchange experiments, we confirmed that dehydrated S-ZrO_2_ predominantly features tridentate SO_4_ groups, while hydration leads to the breaking of one S–O–Zr bond, resulting in bidentate sulfate coordination.

Extending this analysis to UiO-66, we found that sulfation significantly alters the accessibility of Zr sites in defective pores and modifies the framework’s coordination environment. The incorporation of SO_4_ groups in UiO-66 prevents CO adsorption at Zr sites, in contrast to UiO-66-FA, which exhibits accessible Lewis acid sites. However, UiO-66-SO_4_ readily adsorbs acetonitrile, even after room temperature activation, suggesting that sulfate groups modify, but do not completely block, access to Zr centers. A careful comparison of UiO-66 samples with defective pores that were either blocked, free, or sulfated confirmed that SO_4_ groups are primarily located inside defective pores.

By drawing parallels with S-ZrO_2_, we propose that SO_4_ groups in UiO-66-SO_4_ adopt a bidentate coordination mode to the Zr_6_O_6_ clusters. Unlike in S-ZrO_2_, where dehydration can reversibly induce tridentate coordination, this process does not occur in UiO-66-SO_4_ due to the lower thermal stability of the MOF framework. The presence of H-bonded hydroxyls in UiO-66-SO_4_ after activation at 250 °C further supports this model, indicating that SO_4_ groups promote the formation of Zr-OH groups. These sites remain inaccessible to CO due to its weaker basicity, whereas acetonitrile, as a stronger base, can displace OH groups and bind to Zr centers in defect pores.

Overall, our findings provide new insights into the coordination chemistry of sulfated UiO-66 and the role of sulfate groups in modifying adsorption properties. This study contributes to a deeper understanding of defect chemistry in MOFs and suggests that sulfation can serve as a strategy for tuning the acidity and adsorption behavior of UiO-66-based materials, with potential applications in catalysis and adsorption processes.

## Figures and Tables

**Table 1:** Positions of the S–O stretching modes in the IR spectra of some sulfates.

Sample	Coordination	ν_s_, cm^−1^	ν_as,_ cm^−1^				Ref.
Free SO_4_^2−^	no	inactive	1104				[58]
[Co(NH_3_)_6_]SO_4_Br	monodentate	970	1044–1032		1143–1117		[59]
[Cr(H_2_O)_5_SO_4_Cl]·0.5H_2_O		978	1040		1130		[61]
[U(SO_4_)_2_(H_2_O)_7_]·2H_2_O		996	1042		1144		[62]
[Co_2_(NH_3_)_8_(NH_2_)(SO_4_)](NO_3_)_3_	bidentate	995	1170	1060–1050		1105	[59]
Na_2_Co(SO_4_)_2_		970	1220	1080		1160	[63]
Fe^3+^_0.67_Fe^3+^_4_(SO_4_)_6_(OH)_2_·20H_2_O (Ferricopiapite)		993	1213	1019		1125	[64]
Organic sulfates	C-O-(SO_2_)-O-C	1450–1350	1230–1150				[60]

**Table 2:** Positions of bands assigned to CO and CD_3_CN coordinated to Zr^4+^ sites (cm^−1^). “ND” indicates that the band was not detected.

Sample	S-ZrO_2_		UiO-66-BA		UiO-66-FA		UiO-66-SO_4_	
Activation temperature	25 °C	450 °C	25 °C	250 °C	25 °C	250 °C	25 °C	250 °C
CO	ND	2195	ND	ND	ND	2173	ND	ND
CD_3_CN	2300	2305	ND	2300	2303	2303	2303	2300

## Data Availability

Data will be made available upon request.

## Supplementary Materials

The following supporting information can be downloaded at: https://www.mdpi.com/article/10.3390/nano15110779/s1, Table S1: Selected examples of MOFs containing sulfate or sulfite groups and their applications; Table S2: Main results of XRD profile analysis; Table S3: Refined structural data alongside crystallographic reference data from the literature for comparison; Table S4: Particle size was calculated using the Scherrer equation; Figure S1: XRD patterns of the S-ZrO_2_ (a) and UiO-66-SO_4_ (b) samples; Figure S2: IR spectra of S-ZrO_2_ sample (a) and UiO-66 samples (b) in ATR geometry; Figure S3: FTIR spectra of S-ZrO_2_ sample during evacuation from room temperature (brown) up to 470 °C (green); Figure S4: Regions from 1800 to 1000 cm^−1^ (a) and from 4000 to 2500 cm^−1^ (b) of IR spectra measured for UiO-66-SO_4_ sample during the activation process; Figure S5: FTIR spectra recorded after evacuation of UiO-66-BA, UiO-66-FA, and UiO-66-SO_4_ samples at room temperature (a) and at 250 °C (b); Figure S6: FTIR spectra of the S-ZrO_2_–^18^O sample after the introduction of CO (5 mbar), followed by stepwise evacuation. Before the experiment, the sample was evacuated at 25 °C (red) or 450 °C (brown); Figure S7: FTIR spectra of the UiO-66-SO_4_ sample after CO introduction (5 mbar), followed by stepwise evacuation. Before the experiment, the sample was evacuated at 25 °C (a) and 250 °C (b); Figure S8: FTIR spectra of the UiO-66-SO_4_ sample after the introduction of CD_3_CN (5 mbar), followed by stepwise evacuation. Before the experiment, the sample was evacuated at 25 °C (a) and 250 °C (b).
