# Supplementary material for: Probing Acidic and Defective Sites in Sulfated UiO-66 and ZrO2 via Adsorptive FTIR Spectroscopy
Source: Nanomaterials (Basel). 2025 May 22;15(11):779. doi: 10.3390/nano15110779 (PMC12156948; doi:10.3390/nano15110779)
Supplement: Supplementary file 1 [file nanomaterials-15-00779-s001.zip › nanomaterials-3617407-supplementary.pdf]

Probing Acidic and Defective Sites in Sulfated UiO-66 and ZrO_2 via Adsorptive FTIR Spectroscopy

Vera V. Butova [1,2,*], Olga A. Burachevskaia [3], Nikola L. Drenchev [1], Andrei A. Tereshchenko [3] and Konstantin I. Hadjiivanov [1,*]

[1] Institute of General and Inorganic Chemistry, Bulgarian Academy of Sciences, 1113 Sofia, Bulgaria;
ndrenchev@svr.igic.bas.bg
[2] Academy of Biology and Biotechnology, Southern Federal University, 344090 Rostov-on-Don, Russia
[3] The Smart Materials Research Institute, Southern Federal University, 344090 Rostov-on-Don, Russia;
oburachevskaya@sfedu.ru (O.A.B.); antereshenko@sfedu.ru (A.A.T.)
* Correspondence: v.butova@svr.igic.bas.bg (V.V.B.); kih@svr.igic.bas.bg (K.I.H.)

1. MOFs with sulfate or sulfite groups

Table S 1 Selected examples of MOFs containing sulfate or sulfite groups and their applications. Abbreviations: MPTMS – mercaptopropyl trimethoxysilane; MBA – 2-mercaptobenzoic acid; BDC-SO_3H – 2-sulfoterephthalate; BDC-$(SH)_2$ – 2,2′,5-dimercaptoterephthalate; BDC-NH_2 – amino-terephthalic acid.

MOF	Group	Modified species	Application	Ref.
UiO-66	-SO_4^{2-}	Zr_6O_6 in defect pores directly	Gas phase isobutene dimerization	[1]
UiO-66	-SO_3H	Defect pores through MPTMS	CO_2 separation	[2]
UiO-66	-SO_3H	Defect pores through the modulator (MBA)	Acetalization of glycerol with acetone	[3]
UiO-66	-SO_3H	Linker – BDC-SO_3H	Capture of Pb^{2+} at low ppm levels	[4]
UiO-66	-SO_3H	Linker - BDC-$(SH)_2$	Conversion of oleic acid to biodiesel	[5]
UiO-66	-SO_3H	Linker - BDC-SO_3H + BDC-NH_2	CO_2 adsorption	[6]
MOF-808	-SO_4^{2-}	Zr_6O_6 in defect pores directly	Various acid-catalyzed reactions	[7]
NU-1000	-SO_4^{2-}	Zr_6O_6 in defect pores directly	Catalytic activation of benzene-d_6 and toluene	[8]

2. XRD

Table S 2. Main results of XRD profile analysis.

Sample	Space group	Lattice parameters			Agreement factors			
		a, Å	c, Å	V, Å^3	R_p, %	R_{wp}, %	R_{exp}, %	GOF
S-ZrO_2	P 42/nmc (137)	3.5970(8)	5.1841(15)	67.07(3)	3.11	4.17	3.30	1.26
UiO-66-BA	F m-3m (225)	20.7731(4)	20.7731(4)	20.7731(4)	8.98	11.42	5.93	1.93
UiO-66-FA		20.7610(5)	20.7610(5)	20.7610(5)	10.17	13.89	5.94	2.34
UiO-66-SO_4		20.7720(3)	20.7720(3)	20.7720(3)	9.03	11.43	5.93	1.93

Profile R-factor $R_p = \frac{\sum_i |y_i(obs) - y_i(calc)|}{\sum_i y_i(obs)}$

i corresponds to the respective point of the profile,

yi(obs) are observed intensities,

yi(calc) are calculated intensities.

Weighted profile R-factor $R_{wp} = \sqrt{\frac{\sum_i w_i (y_i(obs) - y_i(calc))^2}{\sum_i w_i y_i(obs)^2}}$; $w_i = \frac{1}{\sigma^2[y_i(obs)]}$

experimental R factor $R_{exp} = \sqrt{\frac{\sum_i w_i y_i(obs)^2}{n-p}}$

n is a number of profile points,

p is a number of refined parameters.

Goodness of fit $GOF = \frac{R_{wp}}{R_{exp}}$

Table S 3 Refined structural data alongside crystallographic reference data from the literature for comparison.

Sample	Space group	Lattice parameters			Reference
		a, Å	c, Å	V, Å^3	
S-ZrO_2	P 42/nmc (137)	3.5970(8)	5.1841(15)	67.07(3)	this work
ZrO_2		3.5781	5.1623	66.092	[9]
		3.612	5.212	67.999	[10]
		3.5961	5.177	66.949	[11]
		3.59	5.16	66.503	[12]
UiO-66-SO_4	F m-3m (225)	20.7720(3)	20.7720(3)	20.7720(3)	this work
UiO-66		20.7465	20.7465	20.7465	[13]
		20.75298	20.75298	20.75298	[14]
		20.7589	20.7589	20.7589	[15]

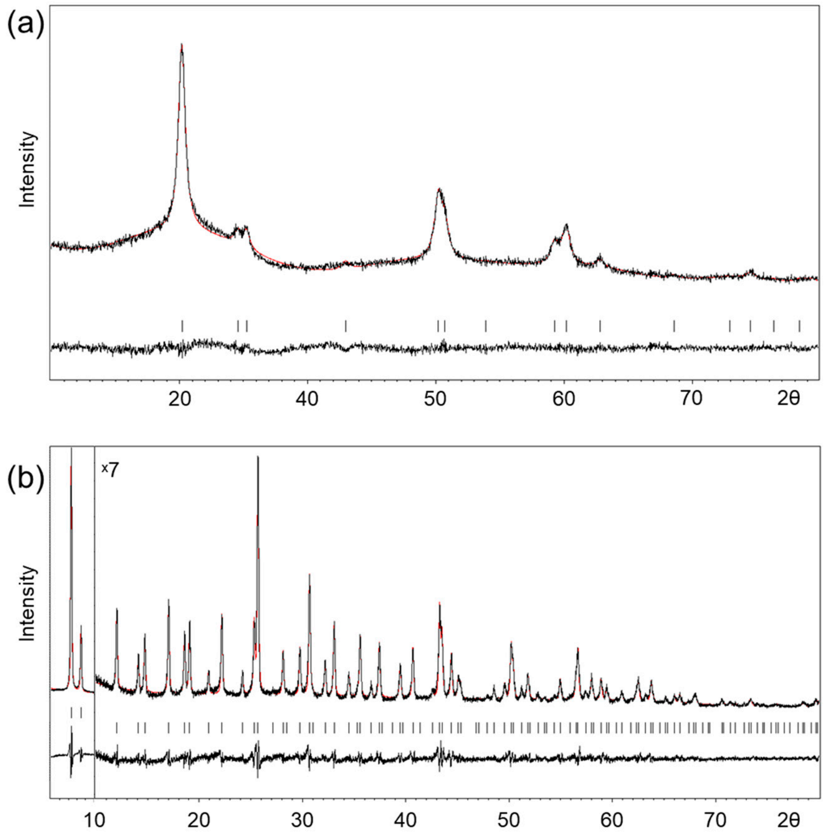

Fig. S1. XRD patterns of the S-ZrO_2 (a) and UiO-66-SO_4 (b) samples. Black profiles represent the experimental data, while red plots correspond to the refined results. The difference between the experimental and calculated data is displayed at the bottom of the figure. Vertical black lines indicate the positions of Bragg peaks. In part (b), the intensity scale beyond 2θ = 10° was multiplied by 7 for better visualization.

Table S 4. Particle size was calculated using the Scherrer equation based on the set of reflections listed in the table. HKL refers to the Miller indices of the reflections. FWHM (Full Width at Half Maximum) values were corrected for instrumental broadening, which was determined as a function of the 2θ angle using a polynomial fit of standard α-quartz plate XRD data.

HKL	Peak position (°2θ)	FWHM (enter as °2θ)	Size (nm)
	S-ZrO_2		
101	30.2157	0.6572	15.57
002	34.574	0.6663	15.42
110	35.2565	0.6678	15.40
200	50.7169	0.7081	14.95
	UiO66-SO_4		
202	12.0407	0.1724	189.38
113	14.1288	0.1736	186.38
222	14.7605	0.1739	185.60
004	17.0598	0.1752	181.33
313	18.6035	0.1761	178.02
204	19.0912	0.1764	176.86

3. Infrared spectroscopy

Bands around 417 cm^{-1} are attributed to Zr–O bonds in ZrO_2, while the band at 1620 cm^{-1} corresponds to water molecules adsorbed on the ZrO_2 surface (Fig. S2a) [16,17].

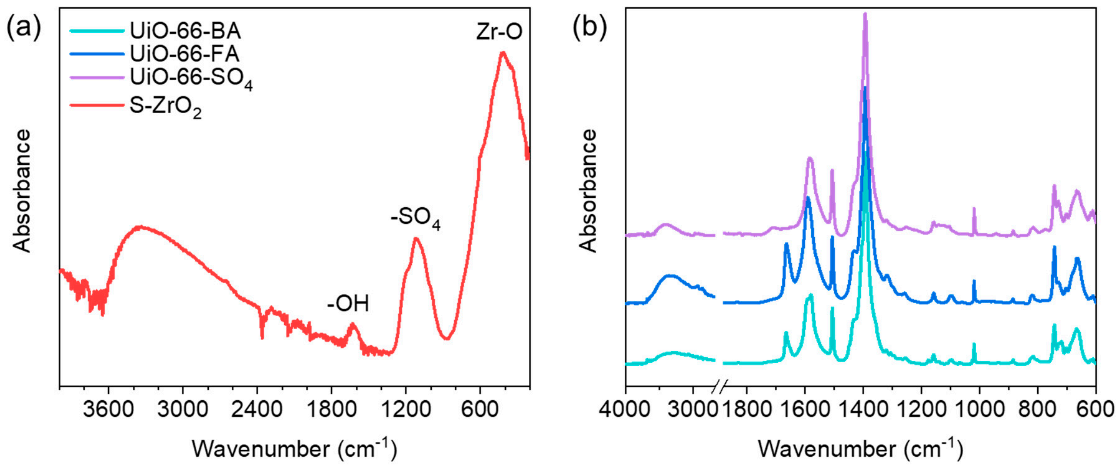

Fig. S2. IR spectra of S-ZrO_2 sample (a) and UiO-66 samples (b) in ATR geometry.

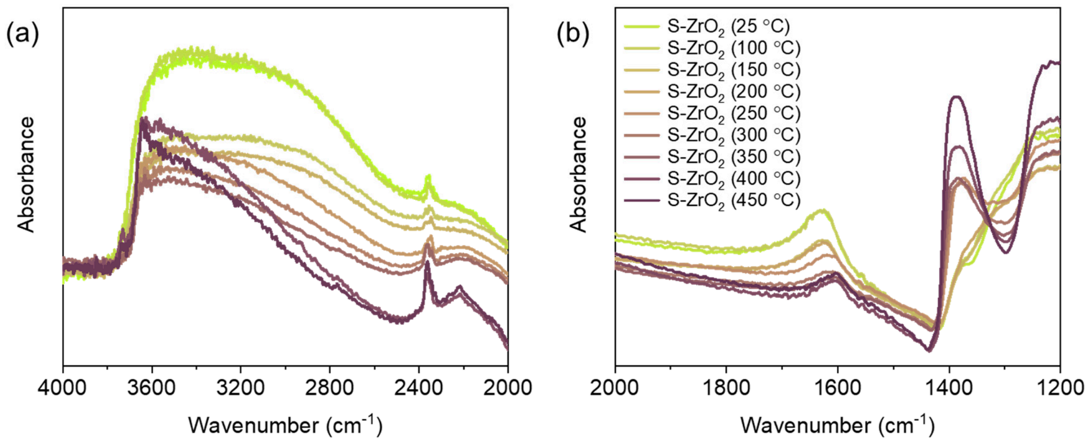

Fig. S3. FTIR spectra of the S-ZrO_2 sample during evacuation from room temperature (brown) up to 470 °C (green). Spectra are provided in the 1800-1200 cm^{-1} (a) and 4000–2000 cm^{-1} (b) regions.

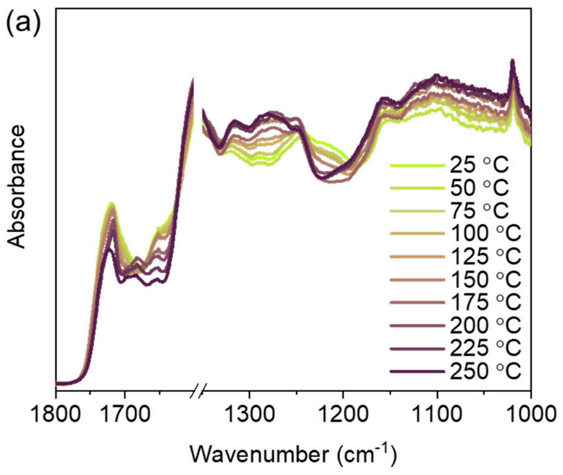

Fig. S4. Regions from 1800 to 1000 cm-1 (a) and from 4000 to 2500 cm-1(b) of IR spectra measured for UiO-66-SO_4 sample during the activation process. Spectra were registered after evacuation at respective temperatures in a dynamic vacuum.

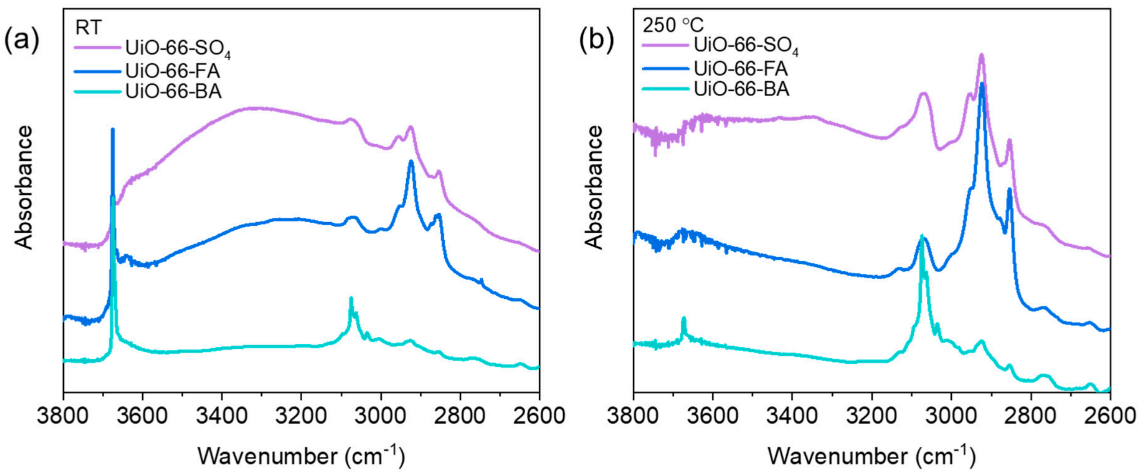

Fig. S5. FTIR spectra recorded after evacuation of UiO-66-BA, UiO-66-FA, and UiO-66-SO_4 samples at room temperature (a) and at 250°C (b).

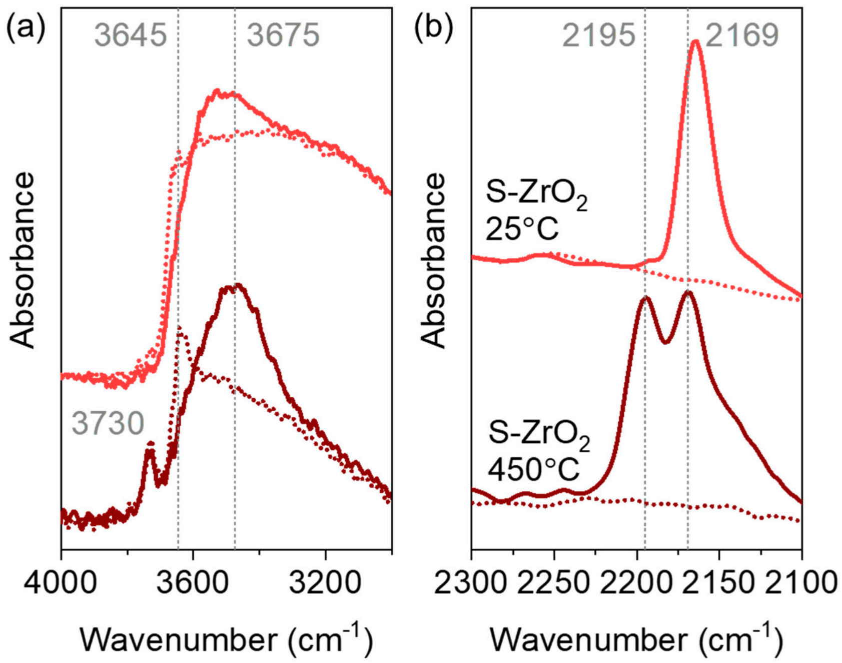

Fig. S6. FTIR spectra of the S-ZrO_2-^{18}O sample after the introduction of CO (5 mbar), followed by stepwise evacuation. Before the experiment, the sample was evacuated at 25 ^{0}C (red) or 450 ^{0}C (brown). Spectra are provided in the regions 4000-3000 cm^{-1} (a) and 2300-2100 cm^{-1} (b).

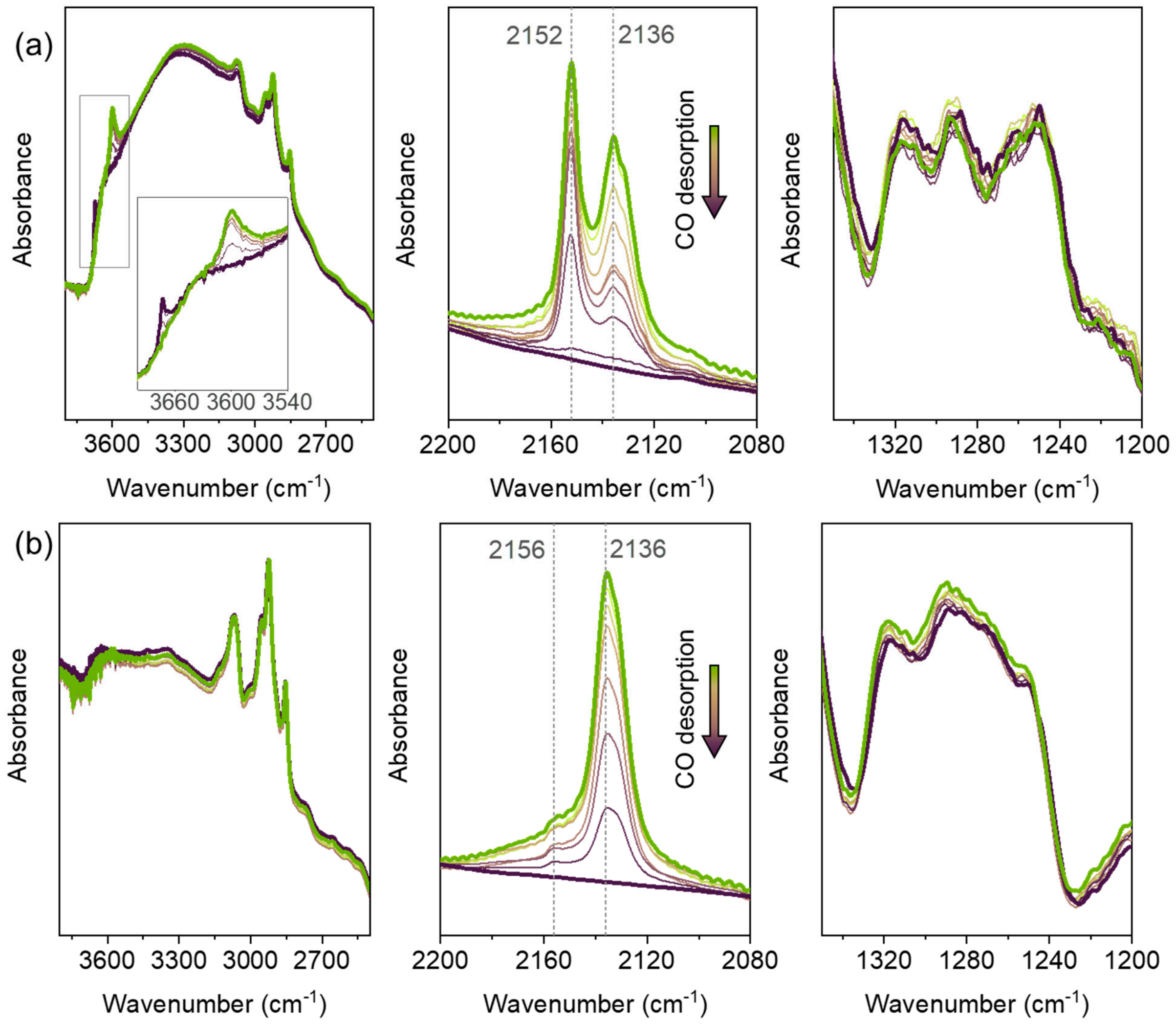

Fig. S7. FTIR spectra of the UiO-66-SO_4 sample after CO introduction (5 mbar), followed by stepwise evacuation. Before the experiment, the sample was evacuated at 25°C (a) and 250°C (b). In part (a), the inset shows a magnified view of the region highlighted by the gray rectangle.

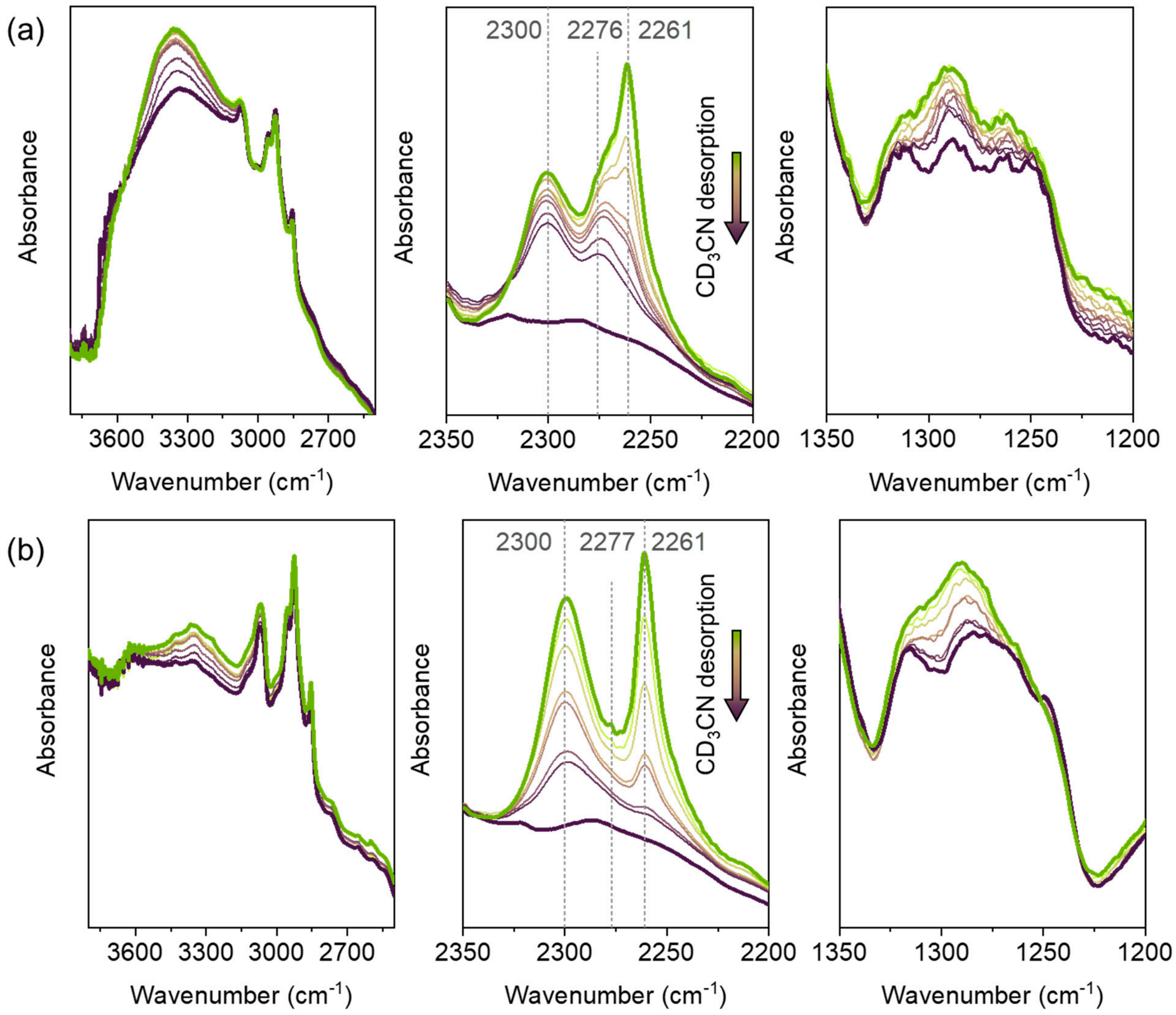

Fig. S8. FTIR spectra of the UiO-66-SO_4 sample after the introduction of CD_3CN (5 mbar), followed by stepwise evacuation. Before the experiment, the sample was evacuated at 25 ^{0}C (a) and 250 ^{0}C (b).

References

1. Fernández-Morales, J.M.; Lozano, L.A.; Castillejos-López, E.; Rodríguez-Ramos, I.; Guerrero-Ruiz, A.; Zamaro, J.M. Direct sulfation of a Zr-based metal–organic framework to attain strong acid catalysts. *Microporous Mesoporous Mater.* **2019**, *290*, 109686. https://doi.org/10.1016/j.micromeso.2019.109686.
2. Tahir, Z.; Aslam, M.; Gilani, M.A.; Bilad, M.R.; Anjum, M.W.; Zhu, L.-P.; Khan, A.L. SO_3H functionalized UiO-66 nanocrystals in polysulfone based mixed matrix membranes: Synthesis and application for efficient CO_2 capture. *Sep. Purif. Technol.* **2019**, *224*, 524–533. https://doi.org/10.1016/j.seppur.2019.05.060.
3. Jiang, Y.; Zhou, R.; Ye, B.; Hou, Z. Acetalization of glycerol over sulfated UiO-66 under mild condition. *J. Ind. Eng. Chem.* **2022**, *110*, 357–366. https://doi.org/10.1016/j.jiec.2022.03.008.
4. Nazari, M.; Amini, A.; Eden, N.T.; Duke, M.C.; Cheng, C.; Hill, M.R. Highly-efficient sulfonated UiO-66(Zr) optical fiber for rapid detection of trace levels of Pb^{2+}. *Int. J. Mol. Sci.* **2021**, *22*, 6053. https://doi.org/10.3390/ijms22116053.

5. Li, H.; Wang, T.; Chu, H.; Rokhum, S.L.; Zhang, Y.; Yu, H.; Xiao, Q.; Guo, M.; Ma, X.; Li, S.; et al. In–situ modification of UiO–66(Zr) organic ligand to synthesize highly recyclable solid acid for biodiesel production. *Chem. Eng. Res. Des.* **2024**, *205*, 713–721. https://doi.org/10.1016/j.cherd.2024.04.040.
6. Raveendran, S.R.D.; Teh, L.P.; Othaman, R.; Chia, C.H. Enhanced carbon dioxide adsorption performance of UiO-66-SO_3H with a mixed ligand strategy. *J. Environ. Chem. Eng.* **2024**, *12*, 113404. https://doi.org/10.1016/j.jece.2024.113404.
7. Jiang, J.; Gándara, F.; Zhang, Y.-B.; Na, K.; Yaghi, O.M.; Klemperer, W.G. Superacidity in sulfated metal–organic framework-808. *J. Am. Chem. Soc.* **2014**, *136*, 12844–12847. https://doi.org/10.1021/ja507119n.
8. Syed, Z.H.; Mian, M.R.; Patel, R.; Xie, H.; Pengmei, Z.; Chen, Z.; Son, F.A.; Goetjen, T.A.; Chapovetsky, A.; Fahy, K.M.; et al. Sulfated zirconium metal–organic frameworks as well-defined supports for enhancing organometallic catalysis. *J. Am. Chem. Soc.* **2022**, *144*, 16883–16897. https://doi.org/10.1021/jacs.2c05290.
9. Bouvier, P.; Djurado, E.; Ritter, C.; Dianoux, A.J.; Lucazeau, G. Low temperature phase transformation of nanocrystalline tetragonal ZrO_2 by neutron and Raman scattering studies. *Int. J. Inorg. Mater.* **2001**, *3*, 647–654. https://doi.org/10.1016/S1466-6049(01)00196-9.
10. Igawa, N.; Ishii, Y. Crystal structure of metastable tetragonal zirconia up to 1473 K. *J. Am. Chem. Soc.* **2001**, *84*, 1169–1171. https://doi.org/10.1111/j.1151-2916.2001.tb00808.x.
11. Lutterotti, L.; Scardi, P. Simultaneous structure and size-strain refinement by the Rietveld method. *J. Appl. Crystallog.* **1990**, *23*, 246–252. https://doi.org/10.1107/S0021889890002382.
12. Bhagwat, M.; Ramaswamy, V. Synthesis of nanocrystalline zirconia by amorphous citrate route: structural and thermal (HTXRD) studies. *Mater. Res. Bull.* **2004**, *39*, 1627–1640. https://doi.org/10.1016/j.materresbull.2004.05.008.
13. Øien, S.; Wragg, D.; Reinsch, H.; Svelle, S.; Bordiga, S.; Lamberti, C.; Lillerud, K.P. Detailed structure analysis of atomic positions and defects in zirconium metal–organic frameworks. *Cryst. Growth Des.* **2014**, *14*, 5370–5372. https://doi.org/doi:10.1021/cg501386j.
14. Butova, V.V.; Aboraia, A.M.; Solayman, M.; Yahia, I.S.; Zahran, H.Y.; Abd El-Rehim, A.F.; Algarni, H.; Khabiri, G.; Soldatov, A.V. The joint effect of naphthalene-system and defects on dye removal by UiO-66 derivatives. *Microporous Mesoporous Mater.* **2021**, *325*, 111314. https://doi.org/10.1016/j.micromeso.2021.111314.
15. Butova, V.V.; Burachevskaya, O.A.; Muratidi, M.A.; Surzhikova, I.I.; Zolotukhin, P.V.; Medvedev, P.V.; Gorban, I.E.; Kuzharov, A.A.; Soldatov, M.A. Loading of the model amino acid leucine in UiO-66 and UiO-66-NH_2: Optimization of metal-organic framework carriers and evaluation of host-guest interactions. *Inorg. Chem.* **2021**, *60*, 5694–5703. https://doi.org/10.1021/acs.inorgchem.0c03751.
